# Association between sleep, care burden, and related factors among family caregivers at home

**DOI:** 10.1111/psyg.12513

**Published:** 2020-01-23

**Authors:** Hirochika Ryuno, Chieko Greiner, Yuko Yamaguchi, Hirokazu Fujimoto, Misato Hirota, Hisayo Uemura, Hitoshi Iguchi, Mai Kabayama, Kei Kamide

**Affiliations:** ^1^ Department of Nursing Kobe University Graduate School of Health Sciences Kobe Japan; ^2^ Social Welfare Corporation Ho‐yu Fukushikai Syownkan Toyono Japan; ^3^ Division of Health Science Osaka University Graduate School of Medicine Suita Japan

**Keywords:** burden of illness, caregivers, community healthcare, long‐term care, sleep

## Abstract

**Aim:**

Several studies have reported a negative correlation between depressive symptoms and family caregivers' (FCs) subjective sleep status. However, there is a paucity of information on the association between objective/subjective sleep status, care burden, and related factors.

**Methods:**

Participants were 23 pairs of care receivers (CRs; M_age_ = 82.7 ± 8.5 years; 69.6% women) receiving long‐term care at home and their FCs (M_age_ = 66.9 ± 11.0 years; 69.6% women). At baseline, demographic data, subjective sleep status (Pittsburgh Sleep Quality Index; PSQI), WHO‐5 well‐being, depressive mood, and frequency of going outdoors were collected. FCs wore a small, wrist‐worn device with an accelerometer to assess objective sleep status for a consecutive 24‐h 2‐week period, and they answered the Zarit Burden Interview short version (ZBI) every night before sleep. After 3 months, CR status was collected and analysed retrospectively.

**Results:**

The mean total sleep time over 2 weeks was 349.5 ± 69.6 min. The mean ZBI score over 2 weeks was 8.8 ± 6.8, which was significantly correlated with total sleep time (r = −0.42; *P* < 0.05), total time in bed (r = −0.44; *P* < 0.05), PSQI (r = 0.62; *P* < 0.01), frequency of going outdoors by CRs (r = −0.42; *P* < 0.05), and WHO‐5 well‐being among CRs (r = −0.50; *P* < 0.05). Multiple regression analyses revealed that total sleep time (β = −0.51; *P* < 0.05) was significantly associated with care burden (adjusted R^2^ = 0.45). At the 3‐month follow‐up, four CRs had been hospitalised or died, and their FCs displayed significantly severe care burden and slept less than at baseline.

**Conclusions:**

Reduced objective total sleep time is significantly associated with the severity of care burden among FCs. Home‐based care is critical in Japan; therefore, it is meaningful to determine how to reduce care burden.

## INTRODUCTION

To provide informal care to a family member compromises caregivers' mental and physical health, including sleep.^1^, ^2^, ^3^, ^4^, ^5^, ^6^ Poor sleep is linked to many other health problems in family caregivers (FCs), such as depression, care burden, and cardiovascular diseases.^1^, ^2^, ^7^, ^8^, ^9^ Although FC sleep has been measured in subjective and objective terms in previous studies, including self‐rated questionnaires to assess perceived sleep, full‐night polysomnography, and actigraphy to assess sleep–wake activity study results have varied depending on the sleep measures used.^1^, ^2^, ^3^, ^4^, ^5^, ^6^, ^7^ Actigraphy seems less influenced by depression than does self‐reported sleep, explaining in part why subjective and objective sleep measures may show little congruency in FCs. However, few studies have investigated the longitudinal association between sleep status, care burden, and related factors measured by actigraphy over a few days.^1^, ^5^ Additionally, few longitudinal studies have investigated the associations of not only FCs' sleep on their care burden, but also the physical and psychosocial characteristics of care receivers (CRs). Therefore, we examined the associations between the sleep status of FCs who provide care at home, their feeling of care burden, and related factors of both FCs and CRs after a 3‐month follow‐up survey.

## METHODS

### Study design and participants

This study was a longitudinal 3‐month follow‐up survey conducted in 2017. CRs were aged 65 years and older, and recipients of the long‐term care insurance system in Japan. We recruited 95 FC and CR dyads from three day‐service offices and one nursing‐home respite service. Of those, 23 dyads receiving long‐term care at home participated. For each dyad, data collection covered 2 continuous weeks. Before admission, participants were excluded if they used nursing‐home respite service, as a night service, during a 2‐week period. After 3 months, all participants were asked to participate in the follow‐up survey about health status of CRs using the same protocol.

### Measurements

Age, sex, level of care required (national standard for care needs was determined by assessing applicants' severity of physical disability and dementia: care levels 1–5, ranging from the lowest to the highest care needs level), CRs' age when started using care services, duration of using care services, and clinical history.^9^, ^10^ FCs and CRs were asked if they had ever been told by a physician that they had dementia, cardiovascular disease, or a fracture (answers coded yes/no). Alcohol consumption was classified into three categories by ethanol units: never, current (1–3 days and <3 units per week), and excessive current (≥3 days and ≥3 units per week). Participants were asked their frequency of going outdoors as a predictor of activities and respite care: once a day, 5–6 days a week, 3–4 days a week, 1–2 days a week, and seldom (housebound).

#### *Sleep measurement*

Objective sleep was measured with the ActiGraph GT9X (ActiGraph, Pensecola, FL, USA), which was worn for 2 consecutive weeks (14 consecutive 24‐h periods) on caregivers' non‐dominant wrist. Actigraphy has been validated in and recommended for use among elderly populations and has been compared favourably with polysomnography, which is deemed the gold standard for sleep assessment.^4^, ^11^, ^12^, ^13^, ^14^ The recorded actigraphy data were analysed using ActiLife software (version 6, by ActiGraph). The following sleep parameters were averaged across the duration: (i) total sleep time (the amount of time spent sleeping in minutes); (ii) total time in bed (the amount of time spent in bed between in‐bed and final out‐of‐bed times of nocturnal sleep period in minutes); (iii) sleep efficiency (ratio of total sleep time to total time in bed); and (iv) wake after sleep onset at night between initial sleep onset and final awakening.

The Japanese version of the Pittsburgh Sleep Quality Index (PSQI) was also used as a baseline assessment to quantify subjective sleep disturbance in FCs.^15^, ^16^, ^17^ PSQI scores range from 0 to 21; scores ≥5 indicate clinically significant sleep disturbances.

#### *Other factor measurements*

FCs' and CRs' well‐being were measured using the Japanese version of the WHO‐Five Well‐being Index (WHO‐5) at baseline.^18^, ^19^, ^20^ The scale consists of five items. The scores range 0–25, and higher scores indicate better well‐being.

Depressive mood was examined using the 15‐item Geriatric Depression Scale (GDS‐15) for both FCs and CRs.^21^ The GDS‐15 was specifically developed to screen and assess depression in the elderly.^21^, ^22^

According to the guidelines of the Japanese Society of Hypertension, FCs measured their blood pressure at home twice in the morning, and the mean of the two measurements was used as morning blood pressure.^23^ FCs used the electronic monitor OMRON HEM‐7325T (OMRON, Tokyo, Japan), and mean blood pressure at home was calculated during the 2‐week period.

### Assessment of care burden

Caregiver burden was assessed with the short version of the Zarit Caregiver Burden Interview (ZBI).^24^, ^25^ It is a commonly used measure of care burden and has been used for the elderly.^24^, ^25^ Total scores range 0–32, and higher scores indicate more severe burden. Every night, FCs completed this questionnaire during the 2‐week period.

### Statistical analyses

Correlations (r) and standardised regression coefficients (β) were used to assess independent associations between variables. In comparison with FCs' characteristics at baseline, data were stratified by CRs' status at follow‐up. Mann–Whitney *U*‐test and Fisher's exact test were used to calculate differences between dropout and follow‐up group. Statistical analyses were performed using SPSS 24 (IBM, Tokyo, Japan). *P* < 0.05 was considered significant.

This study was approved by the internal review board at Graduate School of Health Sciences, Kobe University (no. 544) and was conducted in accordance with the ethical standards laid down in the Declaration of Helsinki. After receiving a complete description of all procedures, all CRs and FCs provided written, informed consent before their inclusion in the study.

## RESULTS

Participants' characteristics are shown in Table 1. Notably, 40.9% of FCs and 56.3% of CRs scored above the GDS‐15 threshold of 5, indicating depression.

**Table 1:** Caregivers' and care receivers' characteristics

	Caregivers (*n* = 23)	Care receivers (*n* = 23)
Age (years)	66.9 ± 11.0	82.7 ± 8.5
Women (%)	69.6	69.6
Stroke (%)	13.6	22.7
Heart disease (%)	22.7	40.9
Hypertension (%)	36.4	40.9
SBP (mmHg)	132.4 ± 19.1	‐
DBP (mmHg)	83.4 ± 10.3	‐
Fracture (%)	27.3	36.4
Dementia (%)	0.0	60.9
ZBI (/32)	8.8 ± 6.8	‐
PSQI (/21)	5.2 ± 3.6	4.5 ± 4.0
PSQI ≥5 (%)	34.8	33.3
WHO‐5 Well‐being Index (/25)	13.8 ± 3.5	15.1 ± 4.5
GDS‐15 (/15)	4.5 ± 3.5	4.7 ± 3.6
GDS‐15 ≥ 5 (%)	40.9	56.3
Alcohol consumption (%)		
Never/current/excessive	45.5/50.0/4.5	78.3/21.7/0.0
Frequency of going outdoors (%)		
Once a day	13.0	0.0
Seldom (housebound)	8.7	39.1

Values are given as means ± standard deviations or percentages.

Scores of 5 or greater on the PSQI indicate clinically significant sleep disturbances.

Abbreviations: SBP, systolic blood pressure; DBP, diastolic blood pressure; ZBI, Zarit Caregiver Burden Interview; PSQI, Pittsburgh Sleep Quality Index; WHO‐5, World Health Organisation‐Five; GDS‐15, Geriatric Depression Scale 15.

Table 2 summarises CRs' utilisation of long‐term care insurance services. Of the in‐home long‐term care services, 87.0% of CRs used day services.

**Table 2:** Utilisation of long‐term care insurance services (*n* = 23)

Care level†	2.5 ± 1.6
Age starting to use care services (years)	77.4 ± 9.3
Duration of using care services (years)	5.2 ± 4.1
Day service (%)	87.0
Home rehabilitation (%)	34.8
Nursing‐home respite service (%)	26.1
Mean days using nursing‐home service per month	6.3 ± 6.0
Visiting nurse (%)	21.7
Home help (%)	21.7
Home bath service (%)	17.4

Values are given as means ± standard deviations or percentages.

† Care levels were determined by assessing applicants' physical and mental status. Assistance required represents five need levels: lowest (care level 1) to highest (care level 5).

FCs' objective and subjective sleep status information and CRs' subjective sleep status information are shown in Tables 1 and 3. FCs experienced frequent night‐time awakenings, and approximately one‐third of FCs and CRs displayed clinically disturbed sleep.

**Table 3:** Objective sleep status of family caregivers (*n* = 23)

Total sleep time (min)	349.5 ± 69.6
Total time in bed (min)	394.7 ± 73.7
Sleep efficiency (%)	88.7 ± 5.4
Wake after sleep onset (min)	45.0 ± 21.8

Values are given as means ± standard deviations.

Table 4 reveals that care burden was significantly correlated with total sleep time, total time in bed, PSQI scores, frequency of going outdoors by CRs, and CRs' subjective well‐being. Other parameters (i.e. GDS‐15 as a predictor of depressive mood) were not correlated with care burden. In the multiple regression model, total sleep time was significantly associated with care burden after adjusting for PSQI, frequency of going outdoors, subjective well‐being, age, and sex among CRs (Table 4).

**Table 4:** Standardised multi‐regression coefficients (β) as predictors of ZBI score (*n* = 23)

Variable	Univariate coefficients (r)	Standardised multi‐regression coefficients (β)
Total sleep time	**−0.42** **	**−0.51** *
Total time in bed	**−0.44** **	‐
PSQI	**0.62** **	−0.06
Frequency of going outdoors†	**−0.42** **	−0.16
WHO‐5 Well‐being Index†	**−0.50** **	−0.52
Adjusted R^2^	‐	0.45

* *P* < 0.05.

** *P* < 0.01.

Bold entries indicate significant values.

The covariates of the model include age and sex of care receivers.

Abbreviations: ZBI, Zarit Caregiver Burden Interview; PSQI, Pittsburgh Sleep Quality Index; WHO‐5, World Health Organisation‐Five.

† Baseline data of care receivers.

Twenty‐three CRs answered the questionnaire at baseline. Of those, four care receivers did not undergo the 3‐month follow‐up survey because of their hospitalisation or death. Table 5 shows the comparison of characteristics at baseline stratifying their CRs' status at the 3‐month follow‐up. ZBI scores of the dropout group were significantly higher than those of the follow‐up group. Further, total sleep time of the dropout group was marginally shorter than that of the follow‐up group. Total time in bed of the dropout group was significantly shorter than that of the follow‐up group. Subjective sleep status (PSQI) of the dropout group was worse than that reported by the follow‐up group. Subjective well‐being of CRs in the dropout group was significantly lower than that reported in the follow‐up group. Depressive mood (GDS‐15) of CRs in the dropout group was higher than in the follow‐up group. Lastly, the level of care required did not significantly differ between dropout and follow‐up groups.

**Table 5:** Comparison of characteristics at baseline by care receivers' status at 3‐month follow‐up (*n* = 23)

	Care receivers' status at follow‐up		
	Dropout (hospitalisation or death)	Follow‐up	*P*
No. of caregivers (*n*)	4	19	
ZBI (/32)	17.9 ± 4.2	6.9 ± 5.6	**0.001**
Total sleep time (min)	290.9 ± 101.6	361.8 ± 57.2	0.062
Total time in bed (min)	312.3 ± 115.6	412.0 ± 50.9	**0.010**
PSQI (/21)	9.8 ± 4.0	4.3 ± 2.7	**0.003**
PSQI ≥5 (%)	75.0	26.3	0.103
WHO‐5 Well‐being Index†	8.3 ± 5.0	16.4 ± 3.1	**0.017**
GDS‐15^†^ (/15)	10.0 ± 4.2	3.9 ± 2.9	**0.019**
Care level†	3.3 ± 1.7	2.3 ± 1.6	0.324

Values are given as means ± standard deviations and percentages.

Bold entries indicate significant values.

Twenty‐three care receivers answered the questionnaire at baseline. Of those, four care receivers did not undergo the 3‐month follow‐up survey because of their hospitalisation or death.

Scores of 5 or greater on the PSQI indicate clinically significant sleep disturbances.

Care levels were determined by assessing applicants' physical and mental status. Assistance required represents five need levels: lowest (care level 1) to highest (care level 5).

Abbreviations: ZBI, Zarit Caregiver Burden Interview; PSQI, Pittsburgh Sleep Quality Index; WHO‐5, World Health Organisation‐Five; GDS‐15, Geriatric Depression Scale 15.

† Baseline data of care receivers.

## DISCUSSION

We examined the association between sleep status of FCs who provide care at home and their subjective and objective feelings of care burden. We revealed that more severe care burden was significantly associated with poorer total sleep time, as measured by ActiGraph; other FCs' characteristics (i.e. total time in bed and PSQI scores) and CRs' characteristics (i.e. frequency going outdoors and subjective well‐being) were not associated in the multi‐regression model.

To the best of our knowledge, this study was the first to demonstrate that an objective measure of total sleep time was associated with FCs' feelings of care burden. A previous study suggested that poorer sleep efficiency among FCs was associated with greater depressive symptoms, whereas, care burden might not be associated with sleep disturbances.^3^ Although there was no association between sleep efficiency and care burden in this study, sleep efficiency was relatively higher than that reported in previous studies.^3^, ^5^ Therefore, shorter total sleep time might be independently associated with feelings of severe care burden in this study.

A significant correlation was observed between FCs' care burden and CRs' frequency of going outdoors. Although total sleep time was significantly associated with care burden rather than these correlated factors of CRs in the multi‐regression model, these findings might help eliminate care burden. Frequency of going outdoors by CRs might predict not only activity and cognitive functioning of CRs, but also no use of respite services. In a previous study, night‐time respite care was associated with significant change in total sleep per night, total time in bed, sleep efficiency, and subjectively rated sleep quality.^6^ Participants in this study were excluded when they used nursing‐home respite service during a 2‐week period (wearing a 24‐h ActiGraph). Additionally, there were no CRs who went outdoors once a day, and more than one‐third of the CRs were housebound; therefore, FCs felt severe care burden and needed more support, especially night‐time respite services, as compared to previous studies. Subjective well‐being of CRs was also correlated with care burden of FCs; that is, to provide care services that meet the needs of CRs was necessary when they did not use nursing‐home respite service.

In this study, FCs who could not participate in the 3‐month follow‐up survey felt more severe care burden, spent less time in bed, and displayed higher PSQI scores at baseline than did FCs in the follow‐up group. When total time in bed was approximately 5 h and FCs perceived severe care burden, it might predict negative health results among CRs, regardless of the level of care that is required. Although we did not clarify the potential factors for hospitalisation or death, it is necessary to consider night‐time respite care when individuals' total time in bed is approximately 5 h, when the total sleep time is less than 5 h, and situations when severe care burden is perceived.

It is noteworthy that this study used 2 consecutive weeks of actigraphy to objectively measure sleep and ZBI scores every night before sleep, while previous studies that reported no significant correlations between sleep and physical/psychological conditions often based their sleep measurements on a few nights of actigraphy or self‐reporting.^1^, ^3^, ^4^, ^5^, ^6^, ^7^ A few nights of actigraphy may not be representative of habitual sleep for FCs. Sleep parameters in this study were considered to indicate the variability of sleep because FCs were affected by night‐time care at home. Additionally, it is necessary to measure not only self‐reported sleep measures, but also objective measurements to assess individuals' sleep status.^1^ Therefore, additional trials with non‐invasive, objectively measured sleep are needed to elucidate the relationships between sleep and physical/psychological conditions.

This study had several limitations. First, the sample size was small for statistical analyses. Additionally, the sex differences reported in a previous study were unclear.^7^ However, this study focused primarily on factors associated with the care burden of FCs as measured during 2 weeks, and the coefficient of determination was relatively higher than that reported in the previous study; therefore, the model was appropriate for identifying the association between care burden and objective sleep.^3^ Second, insomnia diagnosis relies on complaints and subjective sleep quantity estimates; therefore, objective measurement devices, such as polysomnography and actigraphy, do not reliably diagnose insomnia.^13^ Consequently, sleep parameters in this study were representative of the habitual time of sleep. Although the PSQI was used to assess FCs' sleep disturbances, future research on the diagnosis of insomnia that may influence care burden is needed. Third, previous studies considered dementia when analysing the association between care burden and sleep among FCs,^1^, ^2^, ^5^, ^6^, ^9^, ^10^ whereas, in this study, we considered care levels of CRs as determined by assessing applicants' severity of physical disability and dementia. Although care levels of CRs were considered, the severity and symptoms of dementia according to the criteria of the public long‐term care insurance policy and a Diagnostic and Statistical Manual of Mental Disorders 5th edition diagnosis are also necessary. Fourth, we examined 3‐month follow‐up data concerning CRs' status; however, an intensive longitudinal study is needed to determine causal effects. We plan to follow‐up on this research by increasing the number of participants and analysing these relationships using linear mixed models. Although we acquired no data about night‐time service, a previous study suggested the effect of night‐time respite care on the sleep of FCs at home and the difference in FCs' sleep status depending on where they slept.^6^ This might clarify the factors associated with between‐person differences about objective sleep status, that is, objective sleep status of FCs between the days when they used long‐term care services and those when they did not. Additional research is needed to examine the factors that influence FCs' sleep status to clarify the reasons why FCs' sleep differs on days when they used long‐term care services and when they take care of CRs at night. Fifth, because research to date has focused primarily on factors related to negative perceptions, such as care burden and depression, research that identifies factors associated with positive perceptions, such as positive affect and positive aspects of caregiving, is particularly important.^5^ Finally, in consideration of CRs' safety, we did not collect objective sleep status. Although subjective sleep status might predict night‐time care needs, it is also necessary to objectively clarify CRs' sleep status.^26^

Home‐based care is becoming increasingly critical in Japan; therefore, it is meaningful to determine how to reduce objective care burden among FCs.^27^ Our results indicated that reduced objective total sleep time (as measured by the ActiGraph) is significantly associated with FCs' care burden severity. Additionally, care burden severity and a shorter total sleep time might represent more severe care burden among CRs.

## References

[1] Peng HL , Chang YP . Sleep disturbance in family caregivers of individuals with dementia: a review of the literature. Perspect Psychiatr Care 2013; 49: 135–146. 23557457 10.1111/ppc.12005

[2] McCurry SM , Song Y , Martin JL . Sleep in caregivers: what we know and what we need to learn. Curr Opin Psychiatry 2015; 28: 497–503. 26397027 10.1097/YCO.0000000000000205

[3] Beaudreau SA , Spira AP , Gray HL *et al* The relationship between objectively measured sleep disturbance and dementia family caregiver distress and burden. J Geriatr Psychiatry Neurol 2008; 21: 159–165. 18503035 10.1177/0891988708316857

[4] Schwartz J , Allison MA , Ancoli‐Israel S *et al* Sleep, type 2 diabetes, dyslipidemia, and hypertension in elderly Alzheimer's caregivers. Arch Gerontol Geriatr 2013; 57: 70–77. 23522093 10.1016/j.archger.2013.02.008 PMC3696346

[5] Von Känel R , Mausbach BT , Ancoli‐Israel S *et al* Positive affect and sleep in spousal Alzheimer caregivers: a longitudinal study. Behav Sleep Med 2014; 12: 358–372. 24156281 10.1080/15402002.2013.819470 PMC3999303

[6] Lee D , Morgan K , Lindesay J . Effect of institutional respite care on the sleep of people with dementia and their primary caregivers. J Am Geriatr Soc 2007; 55: 252–258. 17302663 10.1111/j.1532-5415.2007.01036.x

[7] Penning MJ , Wu Z . Caregiver stress and mental health: impact of caregiving relationship and gender. Gerontol 2016; 56: 1102–1113. 10.1093/geront/gnv038 26035875

[8] D'Aoust RF , Brewster G , Rowe MA . Depression in informal caregivers of persons with dementia. Int J Older People Nurs 2015; 10: 14–26. 24433320 10.1111/opn.12043

[9] Hirakawa Y , Kuzuya M , Enoki H , Uemura K . Information needs and sources of family caregivers of home elderly patients. Arch Gerontol Geriatr 2011; 52: 202–205. 20399514 10.1016/j.archger.2010.03.019

[10] Onishi J , Suzuki Y , Umegaki H , Nakamura A , Endo H , Iguchi A . Influence of behavioral and psychological symptoms of dementia (BPSD) and environment of care on caregivers' burden. Arch Gerontol Geriatr 2005; 41: 159–168. 16085067 10.1016/j.archger.2005.01.004

[11] Cole RJ , Kripke DF , Gruen W , Mullaney DJ , Gillin JC . Automatic sleep/wake identification from wrist activity. Sleep 1992; 15: 461–469. 1455130 10.1093/sleep/15.5.461

[12] Kushida CA , Chang A , Gadkary C , Guilleminault C , Carrillo O , Dement WC . Comparison of actigraphic, polysomnographic, and subjective assessment of sleep parameters in sleep‐disordered patients. Sleep Med 2001; 2: 389–396. 14592388 10.1016/s1389-9457(00)00098-8

[13] Ancoli‐Israel S , Cole R , Alessi C , Chambers M , Moorcroft W , Pollak CP . The role of actigraphy in the study of sleep and circadian rhythms. Sleep 2003; 26: 342–392. 12749557 10.1093/sleep/26.3.342

[14] Mantua J , Gravel N , Spencer RM . Reliability of sleep measures from four personal health monitoring devices compared to research‐based actigraphy and polysomnography. Sensors 2016; 16: E646. 27164110 10.3390/s16050646 PMC4883337

[15] Buysse DJ , Reynolds CF 3rd , Monk TH , Berman SR , Kupfer DJ . The Pittsburgh sleep quality index for psychiatric practice and research. Psychiatry Res 1989; 28: 193–213. 2748771 10.1016/0165-1781(89)90047-4

[16] Doi Y , Minowa M , Uchiyama M , Okawa M . Development of the Japanese version of the Pittsburgh sleep quality index. Jpn J Psychiatry Treat 1998; 13: 755–763. (in Japanese).

[17] Doi Y , Minowa M , Uchiyama M *et al* Psychometric assessment of subjective sleep quality using the Japanese version of the Pittsburgh sleep quality index (PSQI‐J) in psychiatric disordered and control subjects. Psychiatry Res 2000; 97: 165–172. 11166088 10.1016/s0165-1781(00)00232-8

[18] Psychiatric Research Unit , Mental Health Centre North Zealand. WHO‐Five Well‐being Index (WHO‐5). [Cited 15 September 2016.] Available from URL: https://www.psykiatri-regionh.dk/who-5/Pages/default.aspx

[19] Awata S , Bech P , Yoshida S *et al* Reliability and validity of the Japanese version of the World Health Organization‐five well‐being index in the context of detecting depression in diabetic patients. Psychiatry Clin Neurosci 2007; 61: 112–119. 17239048 10.1111/j.1440-1819.2007.01619.x

[20] Awata S , Bech P , Koizumi Y *et al* Validity and utility of the Japanese version of the WHO‐five well‐being index in the context of detecting suicidal ideation in elderly community residents. Int Psychogeriatr 2007; 19: 77–88. 16970832 10.1017/S1041610206004212

[21] Imai H , Yamanaka G , Ishimoto Y *et al* Factor structures of a Japanese version of the geriatric depression scale and its correlation with the quality of life and functional ability. Psychiatry Res 2014; 215: 460–465. 24388098 10.1016/j.psychres.2013.12.015

[22] Hirosaki M , Ishimoto Y , Kasahara Y *et al* Positive affect as a predictor of lower risk of functional decline in community‐dwelling elderly in Japan. Geriatr Gerontol Int 2013; 13: 1051–1058. 23278960 10.1111/ggi.12008

[23] Japanese Society of Hypertension Committee for Guidelines for the Management of Hypertension . Japanese society of hypertension for the management of hypertension (JSH2014). Hypertens Res 2014; 37: 253–390. 24705419 10.1038/hr.2014.20

[24] Arai Y , Kudo K , Hosokawa T , Washio M , Miura H , Hisamichi S . Reliability and validity of the Japanese version of the Zarit caregiver burden interview. Psychiatry Clin Neurosci 1997; 51: 281–287. 9413874 10.1111/j.1440-1819.1997.tb03199.x

[25] Zarit SH , Reever KE , Bach‐Peterson J . Relatives of the impaired elderly: correlates of feelings of burden. Gerontol 1980; 20: 649–655. 10.1093/geront/20.6.649 7203086

[26] Higami Y , Yamakawa M , Shigenobu K , Kamide K , Makimoto K . High frequency of getting out of bed in patients with Alzheimer's disease monitored by non‐wearable actigraphy. Geriatr Gerontol Int 2019; 19: 130–134. 30463102 10.1111/ggi.13565

[27] Kajiwara K , Mantani A , Noto H , Miyashita M . The relationship between caregiver burden and caregiver pulse rate measured by using a wristwatch‐type pulsimeter with accelerometer in home‐based family caregivers for persons with dementia: pilot study. Psychogeriatrics 2019; 19: 83–84. 30152601 10.1111/psyg.12364

